# Nodular basal cell carcinoma of the scrotum: a case report and review of the literature

**DOI:** 10.3332/ecancer.2024.1789

**Published:** 2024-10-25

**Authors:** Gustavo Hipólito Diaz Infantes, Edgar Fermín Yan Quiroz, Luis Fernando Meza Montoya, José Richard Tenazoa Villalobos

**Affiliations:** 1 Instituto Nacional de Enfermedades Neoplásicas, Lima 15036, Perú; 2 Hospital de Alta Complejidad Virgen de la Puerta – EsSalud, La Esperanza 13013, Perú; 3 Facultad de Medicina, Universidad Privada Antenor Orrego, Trujillo 13008, Perú; 4 Hospital Victor Lazarte Echegaray – Essalud, Trujillo13013, Perú; a https://orcid.org/0009-0001-9261-7088; b https://orcid.org/0000-0002-9128-4760; c https://orcid.org/0000-0003-3622-9408; d https://orcid.org/0009-0005-0465-0670

**Keywords:** skin cancer, male genitalia, scrotum

## Abstract

Basal cell carcinoma (BCC) is the most common non-melanoma type of skin cancer described in humans that originates in the epidermis, more specifically in the basal layer and its appendages. Environmental, genetic and phenotypic factors contribute to the onset of this cancer; however, damage caused by ultraviolet radiation from sunlight is the primary risk factor. The emergence of this neoplasm in unexposed body areas, such as the soles, groin, armpit, scrotum or vulva is very rare. We present this case of a 71-year-old man with a tumour in the middle raphe of the scrotum histologically confirmed as BCC, which was successfully surgically managed.

## Introduction

Basal cell carcinoma (BCC) represents 70% of non-melanoma type malignant skin neoplasms [1] and the incidence is imprecise because it is not correctly registered in many countries [2]; however, it increases as age advances, people between 55 and 75 years old have an incidence up to 100 times more than those of 20 years old. Chronic exposure to ultraviolet rays of the sun is the main associated factor, the type, quantity and time of exposure associated with the risk of BCC is not defined; however, intermittent frequency and increased intensity have a close association; in addition, the exposure in childhood appears to be more important than photoexposure during adult life [3].

However, there are cases of BCC with a rare and unusual location in anatomical sites that are protected from the sun such as the genital area. These neoplasms in the aforementioned area represent less than 1% of all BCC. Furthermore, the aetiology of this type of lesion continues being unknown. Given its extreme rarity, it represents a challenging situation in daily clinical suspicion [4, 5], for which reason we will present the following case report, where the natural history of the disease, physical examination, complementary test, diagnosis, treatment and follow-up are described.

## Case report

**Figure 1.**
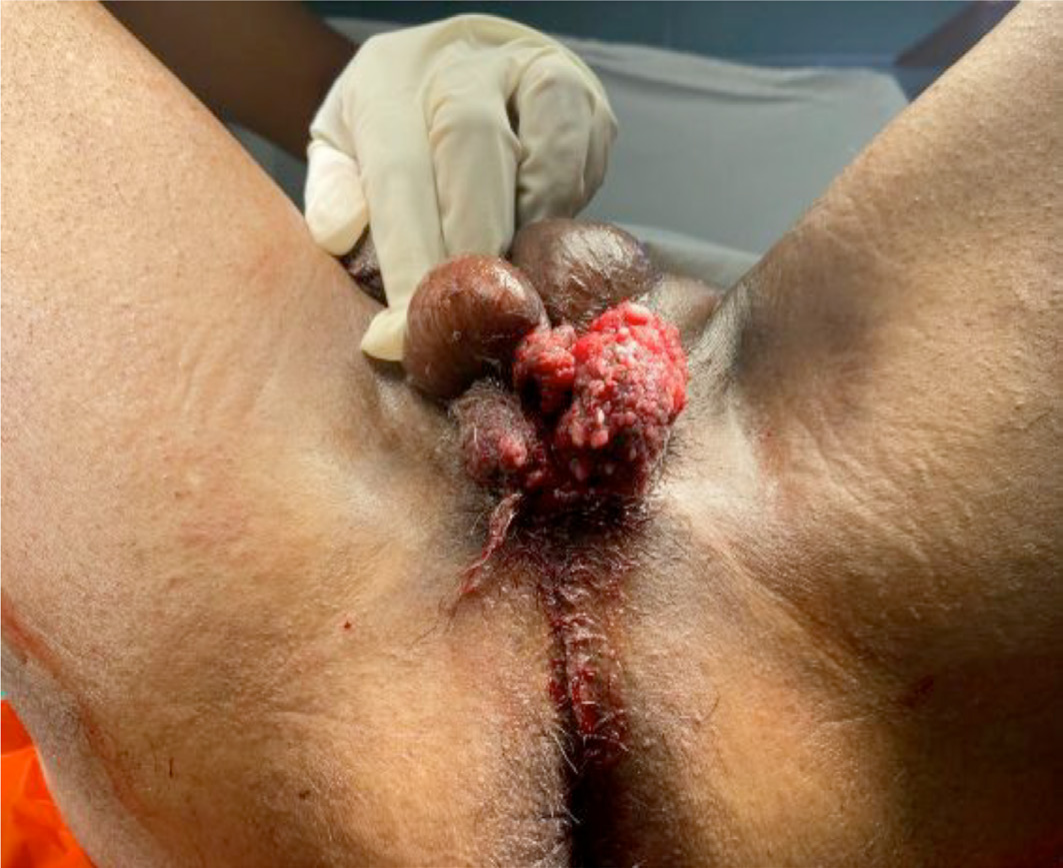
Verrucoid mass in the middle raphe of the scrotum with slight extension towards the left scrotal skin measuring 4 × 3 cm.

Male, 71 years old, with no significant family history of cancer. He has a history of cardiac arrhythmia and smoking, in treatment with bisoprolol 5 mg every 24 hours. He denies sexually transmitted diseases. He was referred to the Oncologic Urology Outpatient Clinic of the National Institute of Neoplastic Diseases because he had been ill for 4 years, where he manifested a progressively growing scrotal lesion. Physical examination of the patient showed an exophytic and verrucoid lesion of approximately 4 × 3 cm in the scrotal level, which exceeds the scrotal median raphe, is partially mobile, and does not seem to infiltrate the penile body (Figure 1). Radiological tests such as abdominopelvic ultrasound and chest X-ray were requested and the results were negative for metastasis.

**Figure 2.**
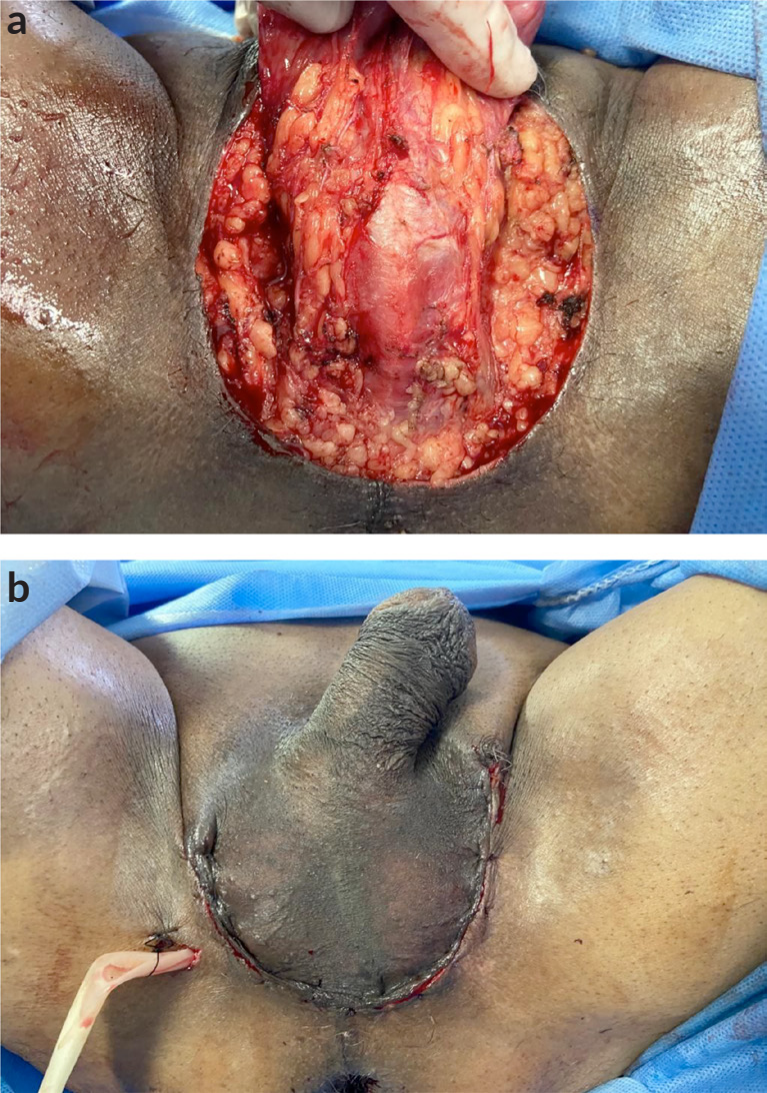
Wide local resection of scrotal lesion. (a): Perineal tissue is exposed and the spermatic cord is preserved, as well as the testicles. (b): Primary skin closure with laminar drain in the right perineum.

**Figure 3.**
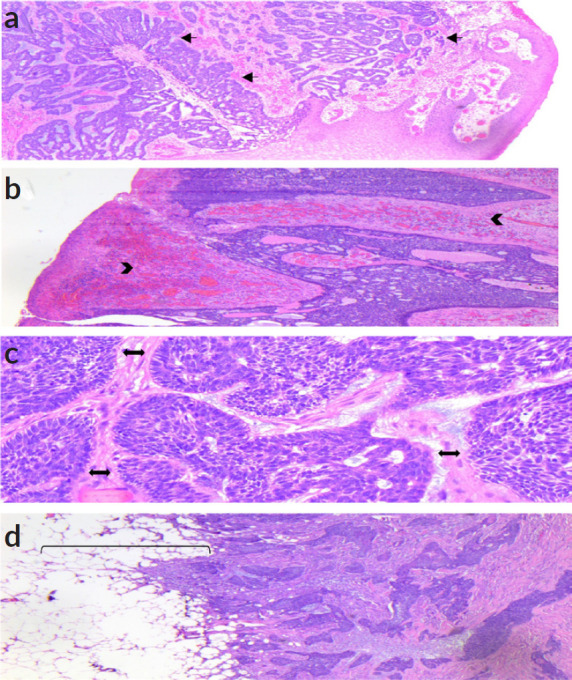
Microscopy. Hematoxylin & Eosin, magnification ×400. (a): Scrotal skin, in which there is basaloid proliferation, with cribriform pattern, in a fibromyxoid stroma (Arrow). (b): The lesion is ulcerated, with haemorrhage and vascular congestion in the stroma (Head arrows). (c): The basaloid proliferation has nests with peripheral palisading (Double ended arrows). (d): The lesion is infiltrative and extends to the adipose tissue (Bracket).

**Figure 4.**
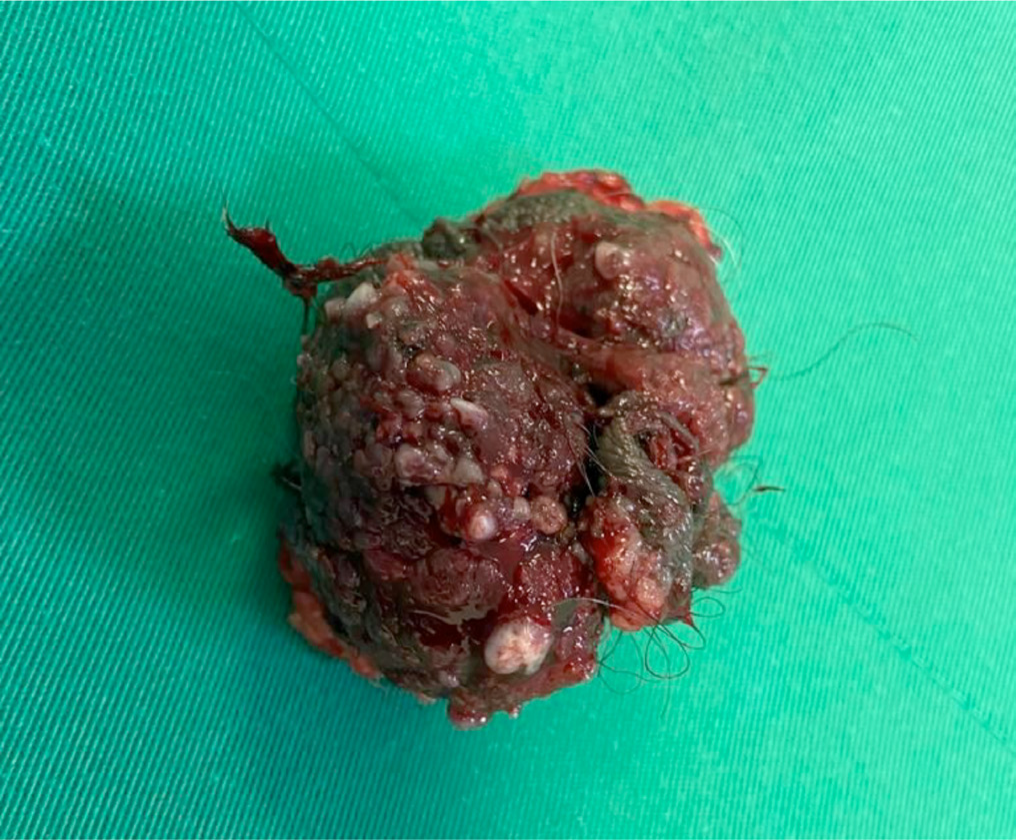
Macroscopy of lesion of the scrotum and perineum. Measurements 6 × 4.5 × 3.7 cm, exophytic with polypoid appearance with ulcerated surface measuring 5 × 4.5 cm.

Due to the extension of the lesion, it was decided to perform a wide local resection of the scrotal lesion plus primary closure and placement of a laminar drain (Figure 2). The patient had a favourable outcome without complications and was discharged 2 days later. The histopathological result revealed: BASOCELLULAR CARCINOMA, growth pattern: nodular and infiltrative; infiltration level: subcutaneous cellular tissue, infiltration depth: 17 mm, perineural invasion: not evident. Surgical edges: free, 5 mm from the deep edge (Figure 3). Macroscopically, the local resection measures 6 × 4.5 × 3.7 cm and the cutaneous surface where an exophytic lesion of polypoid aspect is evidenced, with ulcerated surface measuring 5 × 4.5 and 0.5 cm from the nearest surgical edge, the lesion reaches a depth of 2.5 and 0.5 cm from the surgical edge. It was diagnosed as BCC with a nodular and infiltrative pattern, without perineural invasion (Figure 4).

**Figure 5.**
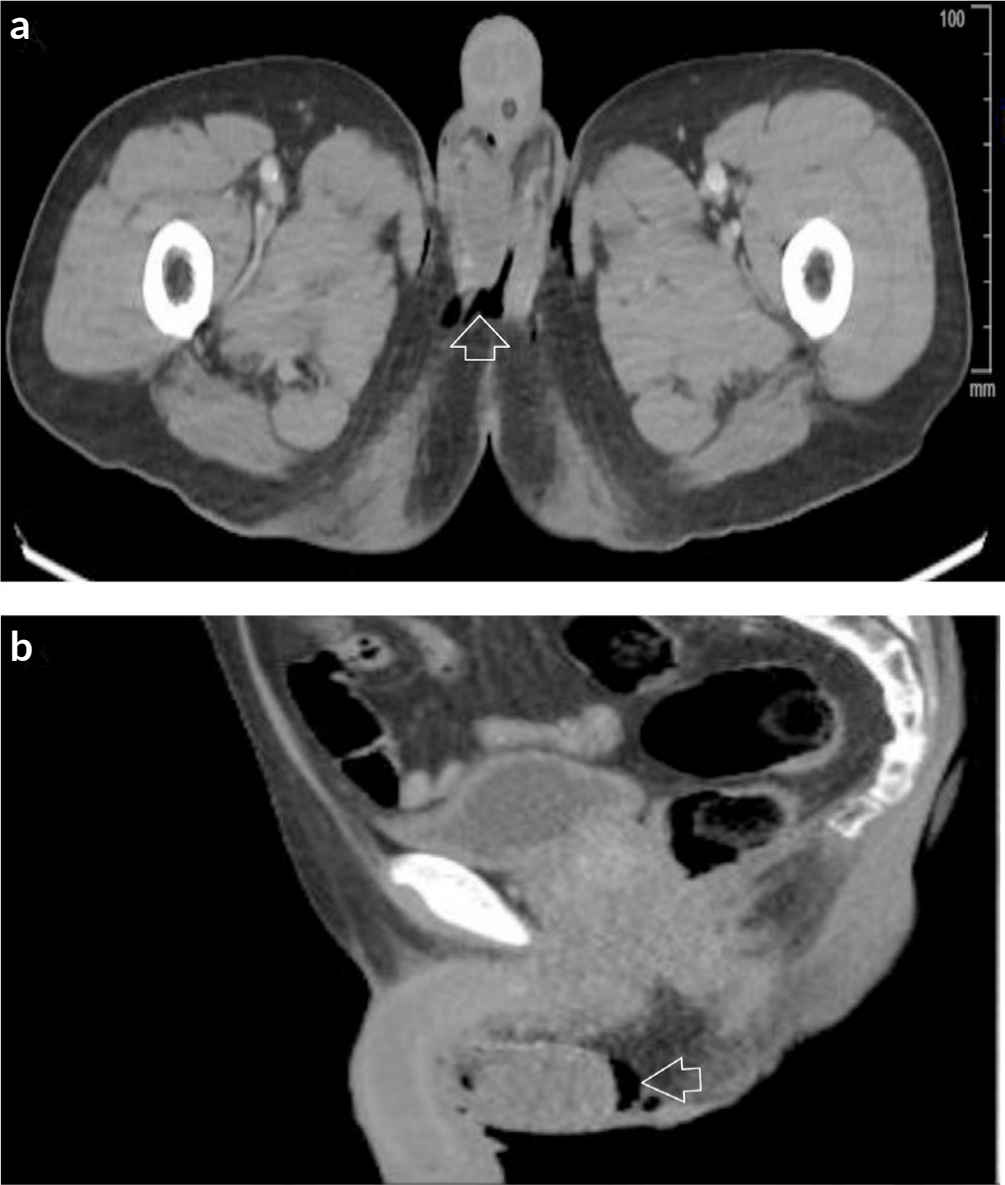
Contrast-enhanced CT scan of the abdomen and pelvis. (a): Axial section. (b): Sagittal view. Post surgical changes at the level of scrotal bags with scrotal emphysema (arrow), no adenopathies or distant metastases are observed.

To rule out the extension of the disease, a computed tomography (CT) scan with intravenous contrast showed no bilateral inguinal and external iliac lymphadenopathy, and emphysema at the level of the scrotal pouches (Figure 5).

The patient attends postoperative clinic controls every 3 months, physical examination shows an unaltered operative wound scar, with no signs of recurrence. No inguinocrural adenopathies are palpable. Currently, 16 months after surgery the patient has not had signs of local recurrence and the urinary and sensory function of the patient is preserved.

## Discussion

BCC is the most common skin neoplasm worldwide and although it arises in skin areas where sun exposure is evident, it can also appear in areas where ultraviolet radiation has no impact such as the genital areas (vulva, scrotum, inguinal and pubic region), palms, soles and axilla. From 2000 to the present, 14 cases of scrotal BCC have been reported in the dermatourological literature [6]. The aetiology of this neoplasia is not defined if it is related to exposure to carcinogens; however, it is known that 40% of p53 gene mutations associated with genetic polymorphisms in loci are present in this malignancy. It has also been proposed that infection of human papillomavirus types 16 and 18 could trigger scrotal BCC [7, 8].

The average age for developing scrotal BCC ranges from the fifth to the seventh decade of life, most of them being nodular or ulcerated. In our case, the patient is in the risk age group and had no personal or family history or previous exposure to ionising radiation or other carcinogens. The behaviour of BCC, regardless of its location, has a low incidence of metastasis (less than 0.1%).

The risk of distant metastasis of this malignant neoplasm of scrotal location can occur in a shorter period and this is explained due to the thin skin of the scrotum with scarce subcutaneous cellular tissue and besides being vastly vascularized [9].

The use of some drugs (tetracyclines, calcium channel blockers, beta-blockers and thiazides) has been documented to generate a modest increase in the risk of BCC. The effect of photocarcinogenic agents, such as furocoumarins that are consumed as citrus products, has also been reported [10, 11].

The nodular type is the most commonly encountered subtype. Macroscopically irregular borders, diffuse telangiectasias and a central ulcer are observed. Wide local resection is the treatment of choice in these cases with high cure rates, they must have free margins of 4 mm and it is imperative to preserve the surrounding tissue; however, the rough contexture of the scrotum makes surgery with free margins difficult. In situations where the resection performed shows a considerable defect, it is recommended to perform a myofasciocutaneous scrotal flap, which is ideal for correcting the genital cutaneous defect. The patient’s expectations must be taken into account, so the considerations must include urinary function, sexual activity and the sociocultural context where they live, especially in young patients; in the case of older patients, this therapeutic plan can be modified [12].

## Conclusion

The case presented was approached surgically with wide local resection plus primary closure, the patient had a well outcome and showed no signs of recurrence in the follow-up after surgery with preserved urological functions. It is feasible in the case of oncologic surgery to completely resect cutaneous malignant neoplasms in non-photoexposed areas such as the scrotal area. Although this field still needs further investigation as to its aetiology, the clinical presentation should be included in the differential diagnosis when evaluating this type of pathology and avoid delays in treatment and subsequent management.

## Conflicts of interest

The authors declare that they have no conflicts of interest associated with the manuscript.

## Funding

This work was self-financed by the authors and received no external funding.

## Ethical approval

Written informed consent was obtained from the patient for publication of this case and the attached images. Approval of the Ethics Committee of the National Institute of Neoplastic Diseases has been obtained.

## Author contributions

GDI and LMM were in charge of the clinical management of the patient and acquired the clinical data, JTV and EYQ drafted the manuscript.
